# Increased levels of ascorbic acid in the cerebrospinal fluid of cognitively intact elderly patients with major depression: a preliminary study

**DOI:** 10.1038/s41598-017-03836-0

**Published:** 2017-06-14

**Authors:** Kenji Hashimoto, Tamaki Ishima, Yasunori Sato, Davide Bruno, Jay Nierenberg, Charles R. Marmar, Henrik Zetterberg, Kaj Blennow, Nunzio Pomara

**Affiliations:** 1 Division of Clinical Neuroscience, Chiba University Center for Forensic Mental Health, Chiba, Japan; 2 Department of Global Clinical Research, Chiba University Graduate School of Medicine, Chiba, Japan; 3 School of Natural Sciences and Psychology, Liverpool John Moores University, Liverpool, UK; 4 Nathan S. Kline Institute for Psychiatric Research, Orangeburg, NY USA; 5 Department of Psychiatry, New York University Langone Medical Center, New York, USA; 6 Clinical Neurochemistry Laboratory, Institute of Neuroscience and Physiology, the Sahlgrenska Academy at the University of Gothenburg, Mölndal, Sweden; 7 Department of Psychiatry and Neurochemistry, Institute of Neuroscience and Physiology, the Sahlgrenska Academy at University of Gothenburg, Mölndal, Sweden; 8 Department of Molecular Neuroscience, UCL Institute of Neurology, London, UK

## Abstract

Major depressive disorder (MDD) in the elderly is a risk factor for dementia, but the precise biological basis remains unknown, hampering the search for novel biomarkers and treatments. In this study, we performed metabolomics analysis of cerebrospinal fluid (CSF) from cognitively intact elderly patients (N = 28) with MDD and age- and gender-matched healthy controls (N = 18). The CSF levels of 177 substances were measured, while 288 substances were below the detection limit. Only ascorbic acid was significantly different, with higher levels in the MDD group at baseline. There were no correlations between CSF ascorbic acid levels and clinical variables in MDD patients at baseline. At the 3-year follow-up, there was no difference of CSF ascorbic acid levels between the two groups. There was a negative correlation between CSF ascorbic acid and CSF amyloid-β_42_ levels in all subjects. However, there were no correlations between ascorbic acid and other biomarkers (e.g., amyloid-β_40_, total and phosphorylated tau protein). This preliminary study suggests that abnormalities in the transport and/or release of ascorbic acid might play a role in the pathogenesis of late-life depression.

## Introduction

Late-life depression, one of the most common psychiatric disorders in older adults, is a growing public health concern as the global population ages. Late-life depression is associated with significant functional impairment, high recurrence rates, chronicity, variable treatment response, and high rates of medical comorbidity and mortality^1–6^. Multiple lines of evidence suggest that late-life depression is a risk factor for the development of mild cognitive impairment and dementia, including Alzheimer’s disease (AD) and vascular dementia^3, 7–10^. However, the precise molecular mechanisms underlying the relationship between late-life depression and dementia risk remain unknown. A precise understanding of this relationship would likely contribute to improving preventive interventions in the elderly.

Metabolomics is the profiling of small molecule metabolites and provides the potential to characterize specific metabolic phenotypes associated with a disease. Metabolomics has an advantage over other “omics” techniques in that it directly samples the metabolic changes in an organism and integrates information from changes at the gene, transcript, and protein levels, as well as posttranslational modifications^10–14^. Cerebrospinal fluid (CSF) is arguably the most relevant sampling substrate for the *in vivo* study of brain disorders as it reflects the metabolic status and the biochemistry of the brain. Metabolomics analyses of CSF in patients and controls therefore have the potential to reveal protein differences linked to the pathogenesis of neuropsychiatric disorders that may have value as biomarkers and might be targets for novel treatments^15–22^. We reported that the CSF ratio of glutamine/glutamate levels in elderly patients with MDD was significantly higher than that of age-matched healthy controls, and that the increased ratio in patients was significantly decreased after 3-year follow-up in association with decreased depression symptoms over this time period, suggesting that abnormalities in the glutamine-glutamate cycle in the brain play a role in the pathogenesis of late-life depression^23^. Furthermore, Pomara *et al*.^24, 25^ reported state-dependent alterations in CSF amyloid-β_42_ levels in cognitively intact elderly MDD patients. In agreement, a recent meta-analysis showed significant reduction of CSF amyloid-β_42_ levels in late-life depression^26^, suggesting that elderly MDD patients have significant differences in amyloid-β metabolism, with a change in CSF amyloid-β_42_ levels in the same direction observed in AD patients. However, there is, currently, no report that has evaluated the metabolomics analysis of CSF of elderly MDD patients.

In the present study, we performed metabolomics analysis of CSF samples from elderly cognitive intact patients with MDD and age- and gender-matched healthy controls. Furthermore, we examined protein expression in postmortem brain samples from controls and psychiatric disorders including MDD patients.

## Results

As reported previously^23–25^, the two groups did not differ on any relevant clinical or demographic variable with the exception of the mean HAM-D score, which as expected was significantly higher in the MDD group (Table 1). Of note, the proportion of participants with a reported family history of AD was slightly higher in the control group than in the MDD group. The CSF levels of amyloid-β_42_ in the MDD group were significantly lower than those of the control group, whereas differences in CSF levels of amyloid-β_40_, total and phosphorylated tau protein did not differ across conditions (Table 1).

**Table 1:** Demographic and Clinical Characteristics of Cognitively Intact Individuals with MDD and Age-Matched Control Subjects at Baseline.

Characteristic	Control Group	MDD Group	Statistical Analysis
	(N = 18)	(N = 28)	P Value
Age (years)	67.3 ± 6.7	66.5 ± 5.4	0.67
Education (years)^a^	16.6 ± 2.8	16.5 ± 2.7	0.86
Body mass index	28.4 ± 4.6	28.8 ± 6.7	0.85
21-item HAM-D	1.1 ± 1.9	14.9 ± 8.8	< 0.001
MMSE	29.5 ± 0.5	29.8 ± 0.6	0.17
Total recall rating	65.1 ± 12.3	64.9 ± 13.9	0.95
Delayed recall rating	8.5 ± 2.9	9.5 ± 2.5	0.24
Trail-Making Test score			
Part A	36.3 ± 12.0	36.0 ± 14.1	0.95
Part B	81.2 ± 32.2	86.1 ± 23.2	0.55
Category fluency test	42.4 ± 7.7	40.6 ± 8.2	0.46
	**N (%)**	**N (%)**	**P Value**
Diabetes	4 (22)	5 (18)	0.72
Female	11 (61)	10 (36)	0.13
Family history of Alzheimer’s disease	5 (28)	3 (11)	0.69
Apolipoprotein genotype			
*APOE e4* positive	4 (22)	11 (39)	
*APOE e4* negative	14 (78)	17 (61)	0.35
	**pg/ml**	**pg/ml**	**P Value**
Amyloid-b_42_	340.2 ± 186.8	224.7 ± 125.1	0.02
Amyloid-b_40_	6374.7 ± 2689.0	5146.0 ± 2369.0	0.11
Total tau protein^b^	311.0 ± 134.3	273.0 ± 114.3	0.31
Phosphorylated tau protein	49.7 ± 19.7	48.9 ± 25.9	0.92

The data are the mean ± standard deviation (SD).

21-item HAM-D: 21-item Hamilton Depression Rating Scale, MMSE: Mini-Mental State Examination.

^a^Data for one control subject were not available.

^b^Data for one MDD patient were not available.

The data at baseline are from Pomara *et al*.^26^.

**Figure 1:**
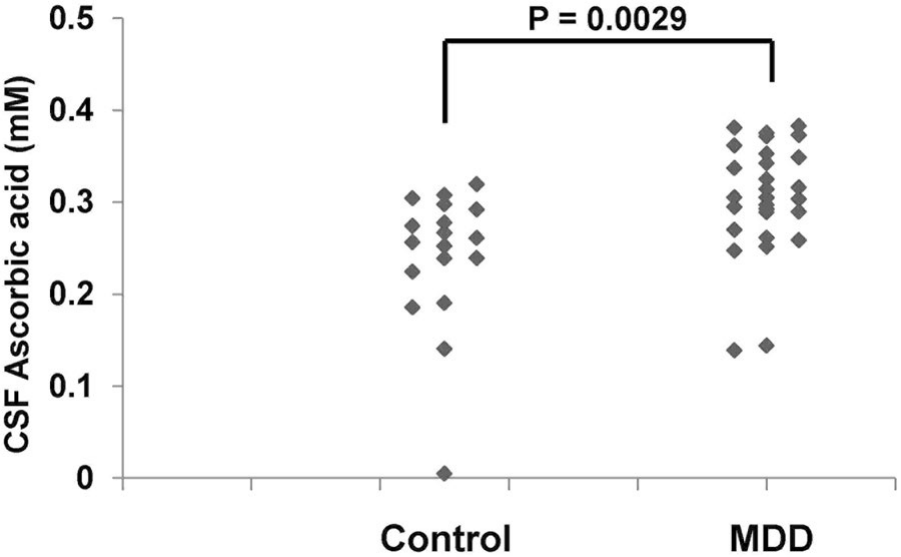
CSF levels of ascorbic acid in control and MDD groups. There was a significant (P = 0.0029) difference in CSF levels of ascorbic acid between the control groups (0.240 ± 0.075 mM, N = 18) and MDD (0.304 ± 0.061 mM, N = 28).

**Figure 2:**
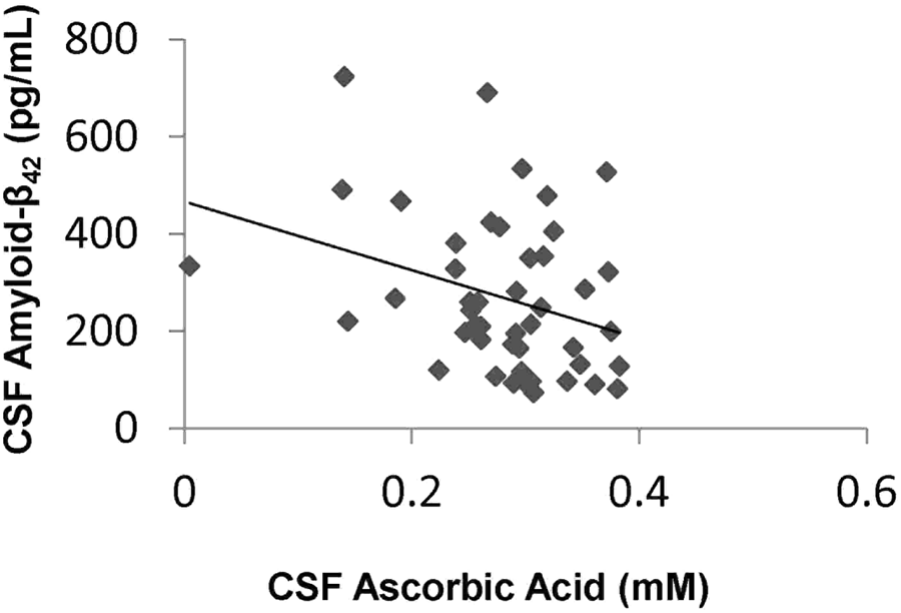
Correlations between CSF ascorbic acid and CSF amyloid-β_42_. There was a significant negative correlation (r = −0.31, P = 0.037) between CSF ascorbic acid and CSF amyloid-β_42_ in the all subjects (N = 46).

We measured 475 major metabolic compounds from various pathways (e.g., glycolytic system, pentose phosphate pathway, citric acid cycle, urea cycle, polyamine-creatine metabolism pathway, purine metabolism pathway, glutathione metabolism pathway, nicotinamide metabolism pathway, choline metabolism pathway and diverse amino acid metabolism pathways). In this study, we were able to measure 177 metabolites, while 288 were below the detection limit. Interestingly, there was a significant (P = 0.0029) difference in CSF levels of ascorbic acid between the MDD (0.304 ± 0.061 mM, N = 28) and the control groups (0.240 ± 0.075 mM, N = 18) (Table S1) (Table 2) (Fig. 1). In addition, the analysis using covariates (BMI, sex, medication) was also statistically significant (P = 0.0037). There were no significant differences in CSF levels of other substances between the MDD and the control groups at baseline (Table S1). At baseline there were no correlations between CSF ascorbic acid levels and clinical variables in the MDD patients (N = 28). No correlations between CSF ascorbic acid levels and other clinical variables were observed. There was a significant negative correlation (Spearman’s r = −0.31, P = 0.037) between CSF ascorbic acid and CSF amyloid-β_42_ in the all subjects (N = 46) (Fig. 2). However, there were no significant correlations between ascorbic acid and other biomarkers (e.g., amyloid-β_40_, total and phosphorylated tau protein) (data not shown). In addition, *APOE e4* did not affect CSF levels of ascorbic acid in control and MDD groups (data not shown).

**Table 2:** HAM-D score and CSF levels of ascorbic acid in subjects at baseline and 3-year follow-up.

Characteristic	Baseline		3-Year Follow-up		Statistical Analyses (P Values)		
	Control (N = 18)	MDD (N = 28)	Control (N = 17)	MDD (N = 19)	ME time	ME MDD	Interaction
21-item HAM-D	1.315 ± 1.565	13.67 ± 8.815	2.235 ± 6.01	8.478 ± 7.79	0.005	<0.001	<0.001
Ascorbic acid (mM)	0.240 ± 0.075	0.304 ± 0.061	0.241 ± 0.095	0.270 ± 0.098	0.175	0.835	0.359

The data are the mean ± standard deviation (S.D.).

MDD: Major depressive disorder, 21 HAM-D: 21-item Hamilton Depression Rating Scale.

Analysis = 2 × 2 Repeated multivariate analysis of variance (MANOVA).

ME time = Main effect of time (baseline and follow up), ME MDD = Main effect of MDD-status (depressed and controls).

At the 3-year follow-up, there was no difference of CSF ascorbic acid levels between two groups although the HAM-D scores in the elderly MDD patients were significantly decreased (Table 2). Furthermore, there were no correlations between ascorbic acid and other biomarkers (e.g., amyloid-β_40_, total and phosphorylated tau protein) (data not shown).

Ascorbic acid (vitamin C) is controlled in the brain parenchyma via the sodium dependent vitamin C transporter, SVCT2, which transfers ascorbic acid at the choroid plexus from blood into CSF, and also from extracellular fluid into neurons^27^. Using Western blot analysis, we measured the expression of SVCT2 in the postmortem samples from the parietal cortex and cerebellum from controls and psychiatric disorders, including MDD, bipolar disorder (BD), and schizophrenia. There was no statistical difference among the four groups (Table 3).

**Table 3:** Expression of SVC2 in the parietal cortex and cerebellum from psychiatric disorders.

Brain regions	Controls	MDD	BD	Schizophrenia	One-way ANOVA
Parietal cortex	1.000 ± 0.052	0.896 ± 0.134	0.907 ± 0.099	0.969 ± 0.078	F (3, 56) = 0.270, P = 0.846
Cerebellum	1.000 ± 0.074	1.117 ± 0.104	0.948 ± 0.084	0.948 ± 0.123	F (3, 56) = 0.661, P = 0.580

The data are the mean ± S.D. (N = 15).

MDD: Major depressive disorder, BD: Bipolar disorder.

The values were shown as the ration of SVCT2 to β-actin.

## Discussion

In the present study, we found that elderly patients with MDD showed increased CSF levels of ascorbic acid compared to age-matched healthy controls, although CSF levels of other metabolites were not different. Furthermore, we found a negative correlation between CSF ascorbic acid and CSF amyloid-β_42_ in the all subjects. Given the possible role of CSF amyloid-β_42_ in the pathogenesis of late-life depression^24, 25, 28–30^, these findings suggest that abnormalities in the brain levels of ascorbic acid might play a role in the depressive symptoms of elderly MDD patients. To our knowledge, this is the first report in the literature of an increase in CSF ascorbic acid in elderly MDD patients, suggesting abnormalities in the transport and/or release of the antioxidant ascorbic acid in the brains of elderly depressed individuals.

Ascorbic acid is a potent water-soluble antioxidant which is not synthesized in the brain. Approximately 40% of ascorbic acid in the brain turns over each day. Ascorbic acid levels are maintained as high as 10 μM in neurons^27, 31^, suggesting that ascorbic acid is crucial for maintenance of oxidative balance. Under conditions of ascorbic acid deficiency, brain content of ascorbic acid is retained tenaciously, with decreases of less than 2% per day^27, 31^. Decreased brain levels of ascorbic acid by deficient diet of ascorbic acid may cause dangerous levels of oxidative stress during normal aging, and particularly during inflammatory neurodegenerative diseases including AD. Thus, brain levels of ascorbic acid are under strong homeostatic regulation^27, 31^. Given the role of oxidative stress and inflammation in late-life depression^32–36^, it is likely that abnormalities in CSF ascorbic acid associated with oxidative stress may play a role in the pathophysiology of late-life depression.

It has been reported that CSF levels of ascorbic acid in humans were higher than blood^37–39^, supporting ascorbic acid as a “nourishing liquor” that constantly surrounds the brain^40^. At the choroid plexus, ascorbic acid is actively transported across the basolateral membrane by SVCT2 into the epithelium and then released into the CSF^31, 39^. In this study, we found higher CSF levels of ascorbic acid in elderly patients with MDD, suggesting that higher CSF levels of ascorbic acid depends on both active “carrier” transport processes and “barrier” integrity of the blood-brain barrier. This may be necessary to prevent ascorbic acid from diffusing out of the brain driven by the concentration gradient^39^. However, we did not find any changes in the expression of SVCT2 in the postmortem brain samples from MDD. Nonetheless, further research on the role of SVCT2 in the transport of ascorbic acid is needed.

It has also been reported that the CSF: plasma ratio of ascorbic acid in AD patients was higher than that in controls^39, 41–43^, suggesting increased consumption of ascorbic acid as a result of oxidative stress in the AD brain, leading to lower plasma levels^39^. In 32 adults followed for one year with mild-to-moderate AD, the rates of cognitive decline were not explained by CSF or plasma ascorbic acid independently^39^. Taken together, it seems that increased CSF levels of ascorbic acid in elderly MDD patients may be due to an increased transport of ascorbic acid into CSF from blood although plasma levels of ascorbic acid were not measured in this study. Further precise studies underlying the reasons of higher CSF levels of ascorbic acid are needed.

A recent study using [^11^C]PK11195 and positron emission tomography demonstrated an increased microglial activation in the brain from patients with late-life depression^44^, suggesting neuroinflammation (or oxidative stress) in the brain in elderly MDD patients. It is well known that peripheral inflammatory substances (e.g., C-reactive protein, interleukin (IL)-8, IL-6, tumor necrosis factor (TNF)-α) are higher in patients with late-life depression^35, 36^. Furthermore, immunological biomarkers, such as vascular endothelial growth factor (VEGF), the chemokine eotaxin, TNF-α, interferon-γ, and macropharge inflammatory protein-1α, are associated with brain structure in late-life depression^36^. Taken together, it is likely that compensatory responses to oxidative stress in the brain of elderly MDD patients may contribute to the increased CSF levels of ascorbic acid although further detailed study on this hypothesis is needed.

We previously reported a reduction of amyloid-β_42_ in the same MDD patients at baseline^24^. Interestingly, we found a negative correlation between CSF ascorbic acid and CSF amyloid-β_42_ in the all subjects, suggesting a close relationship between these two biomarkers. Importantly, CSF ascorbic acid in elderly MDD patients was no longer significantly different from controls after a 3-year period; the loss of significance coincided with reduction in the severity of depressive symptoms, suggesting that abnormalities in CSF ascorbic acid in elderly depression may be state-dependent. Furthermore, Pomara *et al*.^24^ reported higher CSF levels of isoprostane, a biomarker for oxidative stress, in the same patients with MDD. However, there was no correlation between CSF ascorbic acid and CSF isoprostane in all the subjects (data not shown). Taken together, these findings highlight that static indices such as levels of ascorbic acid either in CSF or brain reflect dynamic and brain region specific alterations in the oxidative balance.

Humans have lost the ability to synthesize ascorbic acid^27^. A cross-sectional and prospective study showed that use of ascorbic acid supplement is associated with reduced prevalence and incidence of AD, suggesting the effectiveness of dietary supplementation of ascorbic acid in older adults^45^. A recent meta-analysis showed that serum levels of ascorbic acid in patients with MDD were lower than controls, and that serum levels of ascorbic acid in patients with MDD were increased after antidepressant therapy^46^. Therefore, it is possible that supplementation of ascorbic acid in elderly patients with MDD may contribute to reduced prevalence of AD.

Finally, there are some limitations to this preliminary study that should be noted. The main limitation was small sample size, and similar, future studies in late-life depression would likely benefit from larger sample sizes. In this study, we did not use the multiple test correction since the purpose of this study was the global analysis of a number of metabolites as a pilot study. Follow-up study with much more samples should be conducted to validate our pilot findings. Another limitation was that we did not measure plasma ascorbic acid in the subjects of this study. Given the role of CSF: plasma ratio of ascorbic acid^39, 42, 43^, it is of great interest to study the relationship between CSF: plasma ratio of ascorbic acid and late-life depression.

In conclusion, we found that the CSF levels of ascorbic acid in elderly patients with MDD were significantly higher than that of age-matched healthy controls. These preliminary findings suggest that oxidative imbalance in the brain reflected by abnormalities in CSF ascorbic acid play a role in the pathogenesis of late-life depression. Further studies measuring CSF and plasma levels of ascorbic acid using larger cohorts, particularly cohorts of antidepressant-naïve MDD patients, will be of great interest.

## Materials and Methods

### Participants

This study was approved by the institutional review boards of the Nathan Kline Institute for Psychiatric Research and the New York University School of Medicine. Metabolomics analysis of this study was approved by Research Ethics Committee of the Graduate School of Medicine, Chiba University. All methods were performed in accordance with the guidelines and regulation of the National Institutes of Health, USA. Participants were volunteers who responded to advertisements in local newspapers and flyers or were recruited from the Memory Education and Research Initiative Program^24^. All participants provided informed consent prior to examination and received up to $450.00 in compensation. A total of 133 participants completed the baseline evaluation, and 51 of these took part in the optional lumbar puncture procedure. Of these 51 participants, three were excluded because of evidence in their MRI scans of confluent deep or periventricular white matter hyperintensities, defined as one or more hyperintense lesions measuring at least 10 mm in any direction. One individual was excluded because of a Mini-Mental State Examination (MMSE) score below 28. Of the 47 remaining participants, 28 were diagnosed with MDD by a board-certified psychiatrist, leaving 18 comparison subjects. The structural interview for DSM-IV disorders (SCID) was administered by a psychiatrist to establish an MDD diagnosis. Of the 28 patients with MDD, 21 (75%) had recurrent episodes. Table 1 summarizes the demographic and clinical characteristics of the study participants at baseline.

### Procedure

The study was conducted over four visits, usually one week apart. The first three visits were conducted at the Nathan Kline Institute for Psychiatric Research and the Clinical and Translational Science Institute, New York University Langone Medical Center. During the first visit, for the purpose of obtaining informed consent, study procedures were explained and participants were informed of their rights. Participants’ medical and psychiatric histories, including family history of AD, were also obtained, and their vital signs were measured. Participants then underwent a psychiatric evaluation, and their global cognitive status was assessed using the MMSE. Additionally, the Hamilton Depression Rating Scale (HAM-D) was administered to rate the severity of current depressive symptoms. Subjects who met the criteria for past MDD but were not currently depressed (i.e., HAM-D score below 16) were included as MDD subjects. Blood was drawn for routine clinical labs and *APOE* genotyping. During the second visit, participants underwent an MRI scan of the head to quantify the magnitude of vascular brain pathology. During the third visit, subjects underwent a comprehensive neuropsychological assessment, including the Buschke Selective Reminding Test^47^, the Trail-Making Test, parts A and B^48^, and the category fluency test^49^.

Finally, during the fourth visit, a lumbar puncture was performed by a neuroradiologist under guided fluoroscopy in a subset of participants. Prior to the procedure, which was performed between 9:00 a.m. and 10:00 a.m., participants were asked to fast overnight. A total of 15 ml of clear CSF was collected in three polypropylene tubes labeled “A” (first 5 ml), “B” (second 5 ml), and “C” (third 5 ml). The tubes were immediately placed on ice for a maximum of 1 hour until the samples were centrifuged at 4 °C (at 1500 rpm) for 10 minutes. Then, aliquots of 0.25 ml were placed into 1.00-ml polypropylene cryogenic vials and put into Nunc eight-cell storage boxes (Nalge Nunc International, Rochester, N.Y.) at −80 °C. All amyloid-β, tau, and amino acids determinations were performed from tube “C”.

Among these participants, MDD patients (N = 19) and comparison control subjects (N = 17) were followed for 3 years. The MDD patients were receiving antidepressant, such as including SSRIs (paroxetine, escitalopram, fluoxetine), SNRIs (venlafaxine, duloxetine), and mirtazapine^23, 24^. Clinical data, including physical examination, routine laboratory tests, psychiatric evaluations, HAM-D rating scale, cognitive functions, and CSF samples were collected at 3-year follow-up.

### Metabolomics analysis of human CSF samples

Metabolomics analyses of CSF samples from the MDD and control groups were performed at the Chemicals Evaluation and Research Institute, Japan (CERI, Tokyo, Japan) using an GC-MS/MS based multiple reaction monitoring metabolomics platform. GC/MS/MS analysis was performed using a GCMS-TQ8030 (Shimadzu Co., Kyoto, Japan) with a fused silica capillary column (BPX-5; 30 m × 0.25 mm inner diameter, film thickness: 0.25 µm; SGE Analytical science by Trajan Scientific Australia Pty Ltd). Quantifications of the metabolites were conducted with the built-in GCMS Solution software (Ver.4.20, Shimadzu) and GC/MS/MS Smart Metabolites Database (Ver. 2.0, Shimadzu). In this study, 475 major metabolic compounds from various pathways (glycolytic system, pentose phosphate pathway, citric acid cycle, urea cycle, polyamine-creatine metabolism pathway, purine metabolism pathway, glutathione metabolism pathway, nicotinamide metabolism pathway, choline metabolism pathway and diverse amino acid metabolism pathway) were selected for metabolomics analysis (Table S1).

### Western blot analysis

Postmortem brain samples (parietal cortex and cerebellum) from control, major depressive disorder (MDD), bipolar disorder (BD), and schizophrenia were obtained from the Neuropathology Consortium of the Stanley Medical Research Institute (MD, USA) (Table S2)^50–52^. Tissue samples were homogenized in Laemmli lysis buffer, and total protein levels were measured using the DC protein assay kit (Bio-Rad, Hercules, CA, USA). Aliquots (50 μg of total protein) were incubated for 5 min at 95 °C, with an equal volume of 125 mM Tris/HCl, pH 6.8, 20% glycerol, 0.1% bromophenol blue, 10% β-mercaptoethanol, 4% sodium dodecyl sulfate, and subjected to sodium dodecyl sulfate polyacrylamide gel electrophoresis, using Mini-PROTEAN^®^ TGX Stain-Free™ Gels (AnykD, cat #: 456-8125, Bio-Rad). Proteins were transferred onto polyvinylidenedifluoride (PVDF) membranes using a Trans Blot Mini Cell (Bio-Rad). For immunodetection, the blots were blocked with 3% BSA in TBST (TBS + 0.1% Tween-20) for 1 h at room temperature (RT), and kept with primary antibodies overnight at 4 °C. Membranes were probed using a sodium-ascorbate co-transporter 2 (SVCT2) antibody (cat #: sc-9926, 1: 200, Santa Cruz Biotechnology, CA, USA). The next day, membranes were washed three times in TBST, and incubated with horseradish peroxidase conjugated anti-goat antibody 1 hour, at RT. After final three washes with TBST, the bands were detected using enhanced chemiluminescence (ECL) prime the Western Blotting Detection system (GE Healthcare Bioscience, Tokyo, Japan). The blots then were incubated in the stripping buffer (2% SDS, 100 mM β-mercaptoethanol, 62.5 mM Tris/HCL PH 6.8) for 30 min at 60 °C followed by three time washed with TBST. The stripped blots were kept blocking solution for 1 hour and incubated with the primary antibody directed against β-actin. Images were captured with a Fuji LAS3000-mini imaging system (Fujifilm, Tokyo, Japan), and immunoreactive bands were quantified.

### Statistical analysis

First, Student t-test, and Fisher’s exact test were used to compare the MDD and control groups on MMSE scores, years of education, body mass index, age, incidence of diabetes, gender, *APOE* genotypes, and reported family history of AD (Table 1). Second, Student t-test was used to compare the two diagnostic groups with respect to all remaining CSF variables. Data of 3-year follow-up were analyzed using two-way repeated multivariate analysis of variance (MANOVA). Spearman’s rank correlation coefficient was used. All tests were two-tailed, and statistical significance was established at an α of 0.05, unless differently noted. All analyses were conducted using SPSS 22 (SPSS, Inc., Chicago) and SAS Ver. 9.4 (SAS Institute, Cary, North Carolina, USA).

## Electronic supplementary material

Supplemental information
