# Supplementary material for: Increased levels of ascorbic acid in the cerebrospinal fluid of cognitively intact elderly patients with major depression: a preliminary study
Source: Sci Rep. 2017 Jun 14;7:3485. doi: 10.1038/s41598-017-03836-0 (PMC5471282; doi:10.1038/s41598-017-03836-0)
Supplement: Supplementary file 1 — Supplemental information [file 41598_2017_3836_MOESM1_ESM.pdf]

Supplemental Information

Increased levels of ascorbic acid in the cerebrospinal fluid of cognitively intact elderly patients with major depression: a preliminary study

Kenji Hashimoto,[a] Tamaki Ishima,[a] Yasunori Sato,[b] Davide Bruno,[c] Jay Nierenberg,[d,e] Charles R. Marmar,[e] Henrik Zetterberg,[f,g,h] Kaj Blennow,[f,g] and Nunzio Pomara[d,e]

[a]Division of Clinical Neuroscience, Chiba University Center for Forensic Mental Health, Chiba, Japan, [b]Department of Global Clinical Research, Chiba University Graduate School of Medicine, Chiba, Japan, [c]School of Natural Sciences and Psychology, Liverpool John Moores University, Liverpool, UK, [d]Nathan S. Kline Institute for Psychiatric Research, Orangeburg, NY, USA, [e]Department of Psychiatry, New York University Langone Medical Center, New York, USA, [f]Clinical Neurochemistry Laboratory, Institute of Neuroscience and Physiology, the Sahlgrenska Academy at the University of Gothenburg, Mölndal, Sweden, [g]Department of Psychiatry and Neurochemistry, Institute of Neuroscience and Physiology, the Sahlgrenska Academy at University of Gothenburg, Mölndal, Sweden, and [h]Department of Molecular Neuroscience, UCL Institute of Neurology, London, UK.

Correspondence and requests for materials should be addressed to Dr. Kenji Hashimoto, Division of Clinical Neuroscience, Chiba University Center for Forensic Mental Health, Inohana 1-8-1, Chiba, 260-8670, Japan; TEL: +81-43-226-2517, FAX: +81-43-226-2561 E-mail: hashimoto@faculty.chiba-u.jp

Supplemental Table 1. Metabolomics data of human CSF samples from control subjects and MDD patients.

Peak ID	KEGG ID	Name	Control (n=18, mM)			MDD (n=28, mM)			P-value
			N	Mean	SD	N	Mean	SD	
1	C01026	Dimethylglycine	15	0.00792	0.00183	18	0.00745	0.0016	0.4453
2	C00048	Glyoxylic acid	13	0.00126	0.000248	15	0.00131	0.000195	0.5537
3	C00189	2-Aminoethanol	0	.	.	0	.	.	.
4	C00022	Pyruvic acid	18	0.6117	0.1861	28	0.61	0.0787	0.9656
5	C00186	Lactic acid	18	3.0561	0.4582	28	2.928	0.2955	0.2544
7	-	2-Hydroxyisobutyric acid	18	0.0108	0.00422	28	0.0163	0.0308	0.4606
9	C01585	Caproic acid	18	0.0632	0.0228	28	0.0545	0.0134	0.1098
10	C00160	Glycolic acid	18	0.0661	0.0149	28	0.0667	0.017	0.8975
11	C00041	Alanine	18	0.0465	0.0237	28	0.0407	0.0107	0.2588
12	C00109	2-Ketobutyric acid	17	0.00231	0.00107	27	0.00235	0.000777	0.8799
13	C00141	2-Keto-isovaleric acid	17	0.2166	0.0279	28	0.226	0.0349	0.3505
14	C00192	Hydroxylamine	1	0.00102	.	3	0.0144	0.0069	0.2352
15	C01826	Norvaline	0	.	.	0	.	.	.
18	C05984	2-Hydroxybutyric acid	18	0.0351	0.0131	28	0.0351	0.0113	0.9965
19	C00048	Glyoxylic acid-oxime	10	0.0918	0.0434	19	0.1158	0.0696	0.3328
20	C00209	Oxalic acid	0	.	.	0	.	.	.
23	C00213	Sarcosine	18	0.00915	0.0131	28	0.00736	0.0075	0.5567
24	C00164	Acetoacetic acid	17	0.099	0.0376	24	0.1297	0.0696	0.1066
25	C01013	3-Hydroxypropionic acid	18	0.0383	0.0449	28	0.0271	0.00555	0.1949
26	C03665	2-Aminoisobutyric acid	0	.	.	0	.	.	.
27	C00022	Pyruvic acid-oxime	17	0.00553	0.00342	26	0.00729	0.00356	0.1153
28	C07185	Valproic acid	0	.	.	1	76.5989	.	.
29	C01188	3-Hydroxyisobutyric acid	18	0.1924	0.035	28	0.2083	0.042	0.19
30	-	2-Hydroxyisovaleric acid	18	0.0801	0.0242	28	0.078	0.0254	0.7816
31	C01089	3-Hydroxybutyric acid	11	0.0239	0.0121	16	0.0257	0.0195	0.7908
32	C02261	2-Aminobutyric acid	18	0.0613	0.0292	28	0.0484	0.0162	0.0612
33	C00233	2-Ketoisocaproic acid	4	0.3	0.0637	2	0.2783	0.0201	0.6785
34	C03465	3-Methyl-2-oxovaleric acid	4	0.5461	0.0984	9	0.5418	0.0649	0.926
35	C00407	Isoleucine	1	0.022	.	0	.	.	.
36	-	2-Methyl-3-hydroxybutyric acid	0	.	.	0	.	.	.
37	C00099	3-Aminopropanoic acid	0	.	.	0	.	.	.
38	-	2-Methyl-3-hydroxybutyric acid	0	.	.	0	.	.	.

39	C03465	3-Methyl-2-oxovaleric acid	1	0.00697	.	1	0.0063	.	.
40	C00577	Glyceraldehyde	0	.	.	0	.	.	.
41	C00383	Malonic acid	0	.	.	0	.	.	.
42	C00141	2-Keto-isovaleric acid-oxime	0	.	.	0	.	.	.
43	C00164	Acetoacetic acid	0	.	.	1	0.5405	.	.
45	C05145	3-Aminoisobutyric acid	0	.	.	0	.	.	.
46	C20827	3-Hydroxyisovaleric acid	18	0.119	0.0274	28	0.1153	0.0321	0.6836
47	C00141	2-Keto-isovaleric acid-oxime	0	.	.	0	.	.	.
48	C00183	Valine	18	0.0278	0.0133	28	0.0233	0.00772	0.1515
49	C02170	Methylmalonic acid	0	.	.	0	.	.	.
50	C00233	2-Ketoisocaproic acid	17	1.3566	0.5079	27	1.4489	0.3241	0.4648
51	C00577	Glyceraldehyde	0	.	.	0	.	.	.
52	C00184	Dihydroxyacetone	0	.	.	0	.	.	.
53	C03264	2-Hydroxyisocaproic acid	0	.	.	0	.	.	.
54	C01826	Norvaline	1	0.000923	.	2	0.000929	0.000418	0.9934
55	C00086	Urea	18	6.74	1.8256	28	5.8908	1.8619	0.1354
56	C00164	Acetoacetic acid	0	.	.	1	1.2622	.	.
57	C00184	Dihydroxyacetone	0	.	.	0	.	.	.
59	C00164	Acetoacetic acid-oxime	0	.	.	0	.	.	.
60	C00065	Serine	18	0.0351	0.00936	28	0.0333	0.00739	0.4735
61	C00180	Benzoic acid	18	0.1732	0.2204	28	0.1531	0.1863	0.7404
62	C00189	2-Aminoethanol	18	0.0143	0.00309	27	0.014	0.00177	0.6504
63	C00116	Glycerol	18	0.0265	0.00705	28	0.0224	0.00858	0.103
64	C06423	Octanoic acid	18	0.0361	0.0248	27	0.0236	0.0139	0.0361
65	C00123	Leucine	18	0.0308	0.0138	28	0.0273	0.00866	0.2882
66	C00009	Phosphoric acid	18	0.0179	0.00514	28	0.017	0.00765	0.6632
67	-	Ethylmalonic acid	1	0.000384	.	0	.	.	.
68	C00233	2-Ketoisocaproic acid-oxime	0	.	.	0	.	.	.
69	C00168	3-Hydroxypyruvic acid	0	.	.	0	.	.	.
71	C00407	Isoleucine	18	0.00768	0.00402	28	0.00675	0.0023	0.3215
72	-	Acetylglycine	6	0.0187	0.00661	9	0.0152	0.00613	0.3124
73	C00148	Proline	18	0.00143	0.00138	28	0.00105	0.00063	0.208
74	C00334	4-Aminobutyric acid	0	.	.	0	.	.	.
75	C00037	Glycine	18	0.0133	0.00775	28	0.0105	0.00246	0.0827
76	C01384	Maleic acid	0	.	.	0	.	.	.

77	C00253	Nicotinic acid	0	.	.	0	.	.	.
78	C07086	Phenylacetic acid	0	.	.	0	.	.	.
79	C00042	Succinic acid	7	238348	630610	7	0.000116	0.000114	0.337
80	C00258	Glyceric acid	18	0.0384	0.00964	28	0.0405	0.00533	0.3551
81	C00090	Catechol	0	.	.	0	.	.	.
82	-	Methylsuccinic acid	0	.	.	0	.	.	.
83	C00168	3-Hydroxypyruvic acid	0	.	.	0	.	.	.
84	C00106	Uracil	13	0.000236	0.000038	18	0.00025	0.000132	0.7097
86	C00065	Serine	18	0.0211	0.00558	28	0.0205	0.00472	0.7232
87	C00122	Fumaric acid	3	0.00343	0.00102	5	0.00366	0.000915	0.754
88	C00263	Homoserine	1	0.0188	.	0	.	.	.
89	C01601	Nonanoic acid	18	0.0556	0.0436	28	0.0368	0.0283	0.0828
90	-	Acetylglycine	0	.	.	0	.	.	.
91	C16651	2-Propyl-3-hydroxy-pentanoic acid	0	.	.	0	.	.	.
92	C00188	Threonine	18	0.033	0.0103	28	0.0289	0.00744	0.1315
93	C16651	2-Propyl-3-hydroxy-pentanoic acid	0	.	.	0	.	.	.
94	C00979	O-Acetylserine	0	.	.	0	.	.	.
95	-	Mevalonic lactone	0	.	.	0	.	.	.
96	-	2-Aminooctanoic acid	0	.	.	0	.	.	.
97	C01732	Mesaconic acid	0	.	.	0	.	.	.
98	C00431	5-Aminovaleric acid	8	0.00126	0.000664	6	0.00116	0.000292	0.7353
99	C00178	Thymine	5	1879420	2653810	10	6930881	17205036	0.5327
100	-	Isobutyrylglycine	0	.	.	0	.	.	.
101	C00489	Glutaric acid	0	.	.	1	0.00388	.	.
102	C00530	Hydroquinone	0	.	.	0	.	.	.
103	-	Isobutyrylglycine	0	.	.	0	.	.	.
104	-	3-Methylglutaric acid	0	.	.	0	.	.	.
105	C00099	3-Aminopropanoic acid	0	.	.	0	.	.	.
106	C00263	Homoserine	0	.	.	0	.	.	.
107	C02214	Glutaconic acid	0	.	.	0	.	.	.
108	C02022	Erythrulose	18	0.061	0.0969	28	0.0434	0.0124	0.3458
109	C05145	3-Aminoisobutyric acid	0	.	.	0	.	.	.
110	-	N-Butyrylglycine	0	.	.	0	.	.	.

111	C01571	Decanoic acid	15	0.0592	0.0616	23	0.0321	0.0488	0.141
112	C00036	Oxalacetic acid	0	.	.	0	.	.	.
113	C02022	Erythrulose	0	.	.	0	.	.	.
114	C00815	Citramalic acid	0	.	.	0	.	.	.
115	C16650	2-Propyl-5-hydroxy-pentanoic acid	0	.	.	0	.	.	.
117	C16884	Threitol	18	0.00301	0.00105	28	0.00334	0.00119	0.3556
118	C00429	Dihydrouracil	12	0.0116	0.00307	19	0.0124	0.00286	0.4967
119	C00149	Malic acid	0	.	.	0	.	.	.
120	-	N-Butyrylglycine	0	.	.	0	.	.	.
121	-	2-Aminooctanoic acid	1	0.000031	.	3	0.000043	3.66E-06	0.0959
122	C00503	meso-Erythritol	18	0.0343	0.00586	28	0.0344	0.00698	0.9685
123	C00429	Dihydrouracil	11	0.0122	0.0039	23	0.0119	0.00498	0.8463
124	-	Isovalerylglycine	0	.	.	0	.	.	.
125	C00153	Niacinamide	2	0.000303	0.000093	5	0.0003	0.000142	0.9786
126	C06104	Adipic acid	0	.	.	0	.	.	.
127	-	N-Acetylserine	18	0.0227	0.00566	28	0.023	0.00709	0.8809
128	-	Isovalerylglycine	0	.	.	0	.	.	.
129	-	Glutamic acid 5-methylester	0	.	.	0	.	.	.
130	C00108	Anthranilic acid	2	0.00136	0.00147	2	0.000944	0.00116	0.7819
132	C00049	Aspartic acid	2	0.00859	0.00147	2	0.00721	0.00349	0.6572
133	-	3-Aminoglutaric acid	0	.	.	0	.	.	.
134	C01157	4-Hydroxyproline	18	0.0121	0.0073	28	0.0121	0.00724	0.9852
135	C00073	Methionine	18	0.00409	0.00194	28	0.00374	0.00107	0.4421
136	C00334	4-Aminobutyric acid	3	0.000451	0.000437	3	0.000229	0.000053	0.432
137	C01879	5-Oxoproline	18	0.0271	0.00853	28	0.0243	0.00418	0.1541
138	C00380	Cytosine	0	.	.	0	.	.	.
139	-	3-Methyladipic acid	0	.	.	0	.	.	.
140	C14872	Thiodiglycolic acid	0	.	.	0	.	.	.
141	C01620	Threonic acid	18	0.00181	0.00117	28	0.00166	0.000409	0.5451
142	C01108	Pyrogallol	0	.	.	0	.	.	.
143	C00166	Phenylpyruvic acid	0	.	.	0	.	.	.
144	C16658	2-Propyl-glutaric acid	0	.	.	0	.	.	.
145	C00036	Oxalacetic acid	0	.	.	0	.	.	.
146	C00097	Cysteine	18	0.00246	0.00053	28	0.00257	0.000424	0.4064

147	-	Tiglylglycine	0	.	.	0	.	.	.
148	C20448	5-Hydroxymethyl-2-furoic acid	0	.	.	0	.	.	.
149	C00791	Creatinine	18	0.1216	0.0233	28	0.1231	0.0235	0.828
150	C02630	2-Hydroxyglutaric acid	0	.	.	0	.	.	.
151	-	3-Hydroxyglutaric acid	0	.	.	0	.	.	.
152	-	Succinylacetone	0	.	.	0	.	.	.
153	-	3-Methylcrotonoylglycine	0	.	.	0	.	.	.
154	C02504	2-Isopropylmalic acid	0	.	.	0	.	.	.
155	C00346	O-Phosphoethanolamine	0	.	.	0	.	.	.
156	C00026	2-Ketoglutaric acid	0	.	.	0	.	.	.
157	-	3-Methylcrotonoylglycine	0	.	.	0	.	.	.
158	-	Succinylacetone	0	.	.	0	.	.	.
159	-	Tiglylglycine	0	.	.	0	.	.	.
160	-	3-Phenyllactic acid	3	0.000323	0.00002	2	0.000338	3.07E-06	0.3998
161	-	Succinylacetone	0	.	.	0	.	.	.
162	C01456	Tropic acid	0	.	.	0	.	.	.
163	C00074	Phosphoenolpyruvic acid	0	.	.	0	.	.	.
164	C03761	3-Hydroxy-3-methylglutaric	0	.	.	0	.	.	.
165	-	Succinylacetone	0	.	.	0	.	.	.
166	C00166	Phenylpyruvic acid	0	.	.	0	.	.	.
167	C00026	2-Ketoglutaric acid	0	.	.	0	.	.	.
168	C02656	Pimelic acid	0	.	.	0	.	.	.
169	C00519	Hypotaurine	2	0.00213	0.000226	0	.	.	.
170	-	3-Aminoglutaric acid	0	.	.	0	.	.	.
171	C00077	Ornithine	0	.	.	1	0.017	.	.
173	C00152	Asparagine	0	.	.	0	.	.	.
174	C00302	Glutamic acid	0	.	.	0	.	.	.
175	C06771	Triethanolamine	8	0.000464	0.00032	7	0.00034	0.000133	0.3611
176	C01672	Cadaverine	0	.	.	0	.	.	.
177	C05593	3-Hydroxyphenylacetic acid	0	.	.	0	.	.	.
178	C00026	2-Ketoglutaric acid-oxime	0	.	.	0	.	.	.
179	C00898	Tartaric acid	3	0.000266	0.000043	1	0.00039	.	0.1308
180	C00108	Anthranilic acid	0	.	.	0	.	.	.
181	C00431	5-Aminovaleric acid	0	.	.	0	.	.	.
182	C00476	Lyxose	18	0.00959	0.00185	28	0.0104	0.00181	0.1636

183	C00181	Xylose	18	0.016	0.013	28	0.0234	0.0229	0.2188
184	C00322	2-Ketoadipic acid	1	0.0223	.	3	0.034	0.0114	0.4683
185	C00079	Phenylalanine	18	0.0121	0.00481	28	0.0119	0.00371	0.837
186	C02642	Ureidopropionic acid	1	0.445	.	1	0.731	.	.
187	C00181	Xylose	16	0.0184	0.0151	27	0.0268	0.0243	0.2228
188	C00156	4-Hydroxybenzoic acid	0	.	.	1	0.000062	.	.
190	C00026	2-Ketoglutaric acid-oxime	0	.	.	0	.	.	.
191	C00476	Lyxose	18	0.00923	0.00189	28	0.00992	0.00204	0.2513
192	C00216	Arabinose	18	0.0138	0.00297	28	0.0149	0.00306	0.2409
193	-	Threo-b-hydroxyaspartic acid	0	.	.	0	.	.	.
194	-	Hexanoylglycine	0	.	.	0	.	.	.
195	C00642	4-Hydroxyphenylacetic acid	1	0.00124	.	0	.	.	.
196	C00310	Xylulose	18	0.00192	0.000244	28	0.0019	0.000359	0.8284
197	-	Hexanoylglycine	0	.	.	0	.	.	.
200	C05052	Ribulose	18	0.00162	0.00028	27	0.00177	0.000443	0.2035
201	C02679	Lauric acid	18	0.000744	0.000427	28	0.000635	0.000546	0.4748
202	C00121	Ribose	17	0.00355	0.000673	28	0.00329	0.000788	0.2786
203	C01042	N-Acetylaspartic acid	0	.	.	2	0.002	0.00113	.
204	C00322	2-Ketoadipic acid	0	.	.	0	.	.	.
205	C01042	N-Acetylaspartic acid	0	.	.	2	0.00123	0.000302	.
206	C00155	Homocysteine	0	.	.	0	.	.	.
207	C00152	Asparagine	13	0.000917	0.000636	18	0.000858	0.000341	0.7423
209	C00245	Taurine	0	.	.	1	0.00479	.	.
210	C00606	3-Sulfinoalanine	0	.	.	0	.	.	.
211	-	Ribonolactone	14	0.0028	0.000845	23	0.00265	0.000879	0.6112
212	C00568	4-Aminobenzoic acid	1	0.00574	.	0	.	.	.
213	C02642	Ureidopropionic acid	0	.	.	0	.	.	.
214	C00379	Xylitol	18	0.0106	0.0039	27	0.00952	0.0025	0.2775
215	C01904	Arabitol	18	0.0294	0.00996	28	0.0287	0.00938	0.8296
216	C01684	Rhamnose	5	0.0029	0.000836	10	0.00307	0.00192	0.8532
217	C00661	Glyceraldehyde 3-phosphate	0	.	.	0	.	.	.
218	C00047	Lysine	0	.	.	0	.	.	.
219	C00474	Ribitol	18	0.00358	0.000639	28	0.0034	0.000863	0.4347
220	C08278	Suberic acid	0	.	.	0	.	.	.
221	-	1,6-Anhydroglucose	9	0.0166	0.00917	15	0.0224	0.0176	0.3712

222	C00322	2-Ketoadipic acid-oxime	0	.	.	0	.	.	.
223	C02979	Glycerol 2-phosphate	0	.	.	0	.	.	.
224	C01684	Rhamnose	17	0.012	0.00453	25	0.0113	0.00311	0.5733
225	C01018	Fucose	16	0.0105	0.00233	24	0.00988	0.00289	0.4937
226	C00956	2-Aminoadipic acid	0	.	.	0	.	.	.
227	C00586	2-Deoxy-glucose	0	.	.	0	.	.	.
228	C00661	Glyceraldehyde 3-phosphate	0	.	.	0	.	.	.
229	C00064	Glutamine	18	0.8146	0.4009	28	0.7743	0.3406	0.7164
230	C00322	2-Ketoadipic acid-oxime	0	.	.	0	.	.	.
231	C01018	Fucose	0	.	.	0	.	.	.
232	C00506	Cysteic acid	0	.	.	0	.	.	.
233	C00134	Putrescine	18	0.0031	0.000952	28	0.00279	0.0012	0.3519
234	C00111	Dihydroxyacetone phosphate	0	.	.	0	.	.	.
235	C01685	Ribonic acid	0	.	.	0	.	.	.
236	C03722	Quinolinic acid	0	.	.	0	.	.	.
237	C00417	Aconitic acid	8	0.0106	0.00283	8	0.0092	0.00114	0.2204
238	C00295	Orotic acid	0	.	.	0	.	.	.
239	C00093	Glycerol 3-phosphate	12	0.000215	0.000094	20	0.000258	0.000155	0.4006
240	C00111	Dihydroxyacetone phosphate	0	.	.	0	.	.	.
241	C00430	5-Aminolevulinic acid	0	.	.	0	.	.	.
242	-	2-Phosphoglyceric acid	0	.	.	0	.	.	.
243	C00632	3-Hydroxyanthranilic acid	0	.	.	0	.	.	.
244	C00064	Glutamine	18	0.2693	0.1323	28	0.2728	0.1006	0.919
245	C00586	2-Deoxy-glucose	0	.	.	0	.	.	.
246	C06672	3-Methoxy-4-hydroxybenzoic acid	0	.	.	0	.	.	.
247	C00346	O-Phosphoethanolamine	0	.	.	0	.	.	.
248	C00337	Dihydroorotic acid	0	.	.	0	.	.	.
249	C02637	3-Dehydroshikimic acid	0	.	.	0	.	.	.
250	C00586	2-Deoxy-glucose	0	.	.	0	.	.	.
251	C05582	Homovanillic acid	3	0.00214	0.000378	4	0.00231	0.000692	0.7212
252	C00430	5-Aminolevulinic acid	0	.	.	0	.	.	.
253	C02637	3-Dehydroshikimic acid	0	.	.	0	.	.	.
254	C00597	3-Phosphoglyceric acid	0	.	.	0	.	.	.
255	C00493	Shikimic acid	0	.	.	0	.	.	.

256	C08261	Azelaic acid	0	.	.	0	.	.	.
259	C02037	Glycyl-Glycine	0	.	.	0	.	.	.
260	C00311	Isocitric acid	18	0.3779	0.1518	28	0.3201	0.0736	0.0907
261	-	2-Aminopimelic acid	18	0.0175	0.00851	28	0.0143	0.0036	0.0864
262	C00158	Citric acid	18	0.1315	0.0632	28	0.1071	0.0275	0.0786
263	C00077	Ornithine	18	0.0046	0.00146	28	0.0041	0.0015	0.273
264	C01586	Hippuric acid	0	.	.	0	.	.	.
265	C00230	Protocatechuic acid	0	.	.	0	.	.	.
266	C00062	Arginine	18	0.0263	0.0105	28	0.0245	0.00804	0.5046
267	C00860	Histidinol	0	.	.	0	.	.	.
268	C00795	Tagatose	2	0.00174	0.00206	0	.	.	.
269	C01005	O-Phospho-Serine	0	.	.	0	.	.	.
270	C03758	Dopamine	0	.	.	2	0.0285	0.0122	.
271	C00262	Hypoxanthine	18	0.0015	0.000463	28	0.00173	0.000903	0.3345
272	C02037	Glycyl-Glycine	17	0.2889	0.0856	28	0.2896	0.0612	0.9742
273	C06468	Psicose	18	0.0179	0.0101	28	0.0184	0.0134	0.909
274	C00544	Homogentisic acid	0	.	.	0	.	.	.
275	C01672	Cadaverine	3	0.00556	0.00817	1	0.000523	.	0.6471
276	C00788	Epinephrine	0	.	.	0	.	.	.
277	C06468	Psicose	18	0.0064	0.00191	28	0.00631	0.00162	0.8598
278	C07326	1,5-Anhydro-glucitol	0	.	.	0	.	.	.
279	C00795	Tagatose	18	0.0321	0.00957	28	0.0315	0.00815	0.8054
280	-	Methionine sulfone	0	.	.	0	.	.	.
281	C00764	Sorbose	14	0.2279	0.0694	18	0.2201	0.0604	0.7376
282	-	2-Methylhippuric acid	0	.	.	0	.	.	.
283	C00095	Fructose	18	0.0653	0.0198	28	0.0615	0.0207	0.5395
284	C00250	Pyridoxal	0	.	.	0	.	.	.
285	C00764	Sorbose	4	0.1252	0.0352	10	0.1361	0.033	0.5947
286	C00568	4-Aminobenzoic acid	0	.	.	0	.	.	.
287	C00860	Histidinol	0	.	.	0	.	.	.
288	C02262	Galactosamine	4	0.0705	0.0249	5	0.0665	0.0213	0.8034
289	C01487	Allose	15	0.0173	0.0137	16	0.0113	0.0136	0.231
290	C06424	Myristic acid	18	0.0567	0.0251	28	0.061	0.0617	0.778
291	C00095	Fructose	18	0.1607	0.0486	28	0.1577	0.0416	0.8228
292	C00159	Mannose	18	0.211	0.7113	28	0.0457	0.00963	0.2224

293	C00124	Galactose	1	0.0513	.	0	.	.	.
294	C00632	3-Hydroxyanthranilic acid	0	.	.	0	.	.	.
295	C00944	5-Dehydroquinic acid	14	11.3834	1.7296	18	11.6004	1.2246	0.6806
296	C05584	Vanilmandelic acid	0	.	.	0	.	.	.
297	C00198	Glucono-1,5-lactone	0	.	.	0	.	.	.
298	C00031	Glucose	18	3.5696	0.4552	28	3.6782	0.3489	0.3656
299	C01586	Hippuric acid	18	0.0722	0.0101	28	0.0753	0.00797	0.259
300	C00159	Mannose	18	0.1117	0.0145	28	0.1145	0.0106	0.4488
301	C01551	Allantoin	3	0.000719	0.000148	0	.	.	.
302	C01487	Allose	17	0.0266	0.00546	27	0.0246	0.00666	0.3088
303	C16666	Vanillylamine	0	.	.	0	.	.	.
304	C00279	Erythrose 4-phosphate	2	0.0047	0.000905	2	0.00548	0.0026	0.73
305	C00250	Pyridoxal	0	.	.	0	.	.	.
306	C00388	Histamine	0	.	.	0	.	.	.
307	C02727	N6-Acetyllysine	0	.	.	0	.	.	.
308	C00279	Erythrose 4-phosphate	0	.	.	0	.	.	.
309	C00124	Galactose	0	.	.	0	.	.	.
310	C00147	Adenine	0	.	.	0	.	.	.
311	C02727	N-Acetyl-Lysine	11	1.1762	0.1885	25	1.1681	0.1379	0.8859
312	C00031	Glucose	18	3.8177	0.5461	28	3.9551	0.4411	0.353
313	C03672	4-Hydroxyphenyllactic acid	18	12.937	3.1457	27	14.011	4.7422	0.4036
314	C00314	Pyridoxine	0	.	.	0	.	.	.
315	C00944	5-Dehydroquinic acid	2	0.2231	0.00433	0	.	.	.
316	C00329	Glucosamine	0	.	.	0	.	.	.
317	C08277	Sebacic acid	0	.	.	0	.	.	.
318	C00392	Mannitol	18	0.00245	0.004	28	0.00396	0.00488	0.2794
319	-	N-Acetylglutamine	13	0.1352	0.0612	20	0.1177	0.0429	0.3392
320	C00794	Sorbitol	17	0.0184	0.00837	23	0.0152	0.0091	0.261
321	C01697	Galactitol	9	0.0347	0.0465	14	0.0338	0.0417	0.9624
322	C00047	Lysine	18	0.0438	0.0122	28	0.0397	0.0101	0.2196
323	C01179	4-Hydroxyphenylpyruvic acid	0	.	.	0	.	.	.
324	C00191	Glucuronic acid	1	0.1762	.	0	.	.	.
325	C03107	Glucono-1,4-lactone	0	.	.	0	.	.	.
326	-	2-Methylhippuric acid	0	.	.	0	.	.	.
327	C00483	Tyramine	4	0.00273	0.00299	1	0.00093	.	0.6275

328	C00333	Galacturonic acid	0	.	.	0	.	.	.
329	C00135	Histidine	4	0.000144	0.000148	0	.	.	.
330	C00329	Glucosamine	0	.	.	0	.	.	.
331	-	3,4-Dihydroxybenzylamine	0	.	.	0	.	.	.
332	C00785	Urocanic acid	0	.	.	0	.	.	.
333	C00191	Glucuronic acid	15	0.00105	0.000337	15	0.000961	0.000467	0.5526
334	C00438	Ureidosuccinic acid	0	.	.	1	0.0126	.	.
335	C02262	Galactosamine	0	.	.	0	.	.	.
**337**	**C00072**	**Ascorbic acid**	**18**	**0.2405**	**0.0751**	**28**	**0.3044**	**0.0615**	**0.0029**
338	C00082	Tyrosine	18	0.00487	0.00201	28	0.00486	0.00123	0.9928
339	C00534	Pyridoxamine	0	.	.	0	.	.	.
340	C00333	Galacturonic acid	5	0.0199	0.00648	5	0.0386	0.0402	0.3351
341	-	N-Acetylglutamine	0	.	.	0	.	.	.
342	C02727	N6-Acetyllysine	0	.	.	0	.	.	.
343	C00788	Epinephrine	7	0.0863	0.0233	7	0.1164	0.0862	0.3895
344	C00590	Coniferyl alcohol	0	.	.	0	.	.	.
345	C07588	2-Hydroxyhippuric acid	0	.	.	0	.	.	.
346	C00954	Indol-3-acetic acid	0	.	.	0	.	.	.
347	C00823	1-Hexadecanol	1	0.0118	.	1	0.0057	.	.
348	C00437	N-Acetyl-Ornithine	0	.	.	0	.	.	.
349	C02666	Coniferyl aldehyde	0	.	.	0	.	.	.
350	C00257	Gluconic acid	18	0.00196	0.000438	28	0.00212	0.0012	0.5995
351	C02666	Coniferyl aldehyde	0	.	.	0	.	.	.
352	C00398	Tryptamine	4	0.000967	0.000276	8	0.00113	0.00042	0.5066
353	C00954	Indol-3-acetic acid	5	0.00983	0.00297	3	0.00923	0.00281	0.7856
354	C00864	Pantothenic acid	7	0.00136	0.000748	13	0.00153	0.000671	0.6133
355	C00725	Lipoic acid	0	.	.	0	.	.	.
356	C00818	Glucaric acid	18	0.000107	0.000029	27	0.000103	0.000025	0.6617
357	C13747	ParaXanthine	0	.	.	0	.	.	.
358	-	S-Benzyl-Cysteine	0	.	.	0	.	.	.
359	C04227	Octopamine	0	.	.	0	.	.	.
360	C00385	Xanthine	16	0.000308	0.000141	23	0.000265	0.000093	0.2564
361	C00785	Urocanic acid	1	0.000136	.	0	.	.	.
362	C08362	Palmitoleic acid	0	.	.	0	.	.	.
363	C00137	Inositol	18	0.1548	0.0343	28	0.1413	0.0311	0.1729

364	C00249	Palmitic acid	18	0.027	0.0121	28	0.0304	0.0234	0.5751
365	C00645	N-Acetylmannosamine	0	.	.	0	.	.	.
367	C03758	Dopamine	4	0.000599	0.00006	8	0.0008	0.000382	0.3315
368	C00199	Ribulose 5-phosphate	0	.	.	0	.	.	.
369	C00117	Ribose 5-phosphate	0	.	.	0	.	.	.
370	C00645	N-Acetylmannosamine	8	0.0441	0.0173	8	0.0376	0.0132	0.414
371	C00117	Ribose 5-phosphate	0	.	.	0	.	.	.
372	C01551	Allantoin	0	.	.	0	.	.	.
374	C00355	DOPA	0	.	.	0	.	.	.
375	C00199	Ribulose 5-phosphate	0	.	.	0	.	.	.
376	C07588	2-Hydroxyhippuric acid	0	.	.	0	.	.	.
377	C02678	Dodecanedioic acid	0	.	.	0	.	.	.
378	-	N-Acetyltyrosine	0	.	.	0	.	.	.
379	C01717	Kynurenic acid	0	.	.	0	.	.	.
380	C00366	Uric acid	18	0.00975	0.0118	28	0.00461	0.00416	0.0395
381	C07202	Metoprolol	0	.	.	0	.	.	.
382	C00534	Pyridoxamine	0	.	.	0	.	.	.
383	C00327	Citrulline	0	.	.	0	.	.	.
384	-	Methoprene acid	0	.	.	0	.	.	.
385	C00242	Guanine	0	.	.	0	.	.	.
386	C00547	Norepinephrine	1	0.00212	.	0	.	.	.
387	-	N-Acetyltyrosine	0	.	.	0	.	.	.
388	-	Margaric acid	13	0.00242	0.00278	14	0.00487	0.00867	0.3392
389	C00328	Kynurenine	0	.	.	0	.	.	.
390	-	Octadecanol	1	0.000532	.	0	.	.	.
391	C03339	2,3-Bisphosphoglyceric acid	0	.	.	0	.	.	.
394	C00542	Cystathionine	0	.	.	0	.	.	.
396	-	Cystamine	0	.	.	0	.	.	.
397	C02242	7-Methylguanine	0	.	.	0	.	.	.
398	C01595	Linoleic acid	0	.	.	0	.	.	.
399	C00712	Oleic acid	9	0.00094	0.00153	13	0.00116	0.00127	0.7152
400	C00078	Tryptophan	18	1.3565	0.9143	27	1.0581	0.5211	0.1704
401	-	Suberylglycine	0	.	.	0	.	.	.
403	C01712	Elaidic acid	2	0.0088	0.00135	1	0.00979	.	0.6558
404	C00315	Spermidine	0	.	.	0	.	.	.

405	C00398	Tryptamine	0	.	.	0	.	.	.
406	C01530	Stearic acid	8	0.0125	0.00681	12	0.0228	0.0149	0.0858
407	C05001	Fructose 1-phosphate	0	.	.	0	.	.	.
408	C05001	Fructose 1-phosphate	0	.	.	0	.	.	.
409	C00085	Fructose 6-phosphate	0	.	.	0	.	.	.
410	C00275	Mannose 6-phosphate	0	.	.	0	.	.	.
411	-	Suberylglycine-2TMS	0	.	.	0	.	.	.
412	C00092	Glucose 6-phosphate	0	.	.	0	.	.	.
413	C00491	Cystine	0	.	.	0	.	.	.
414	C00275	Mannose 6-phosphate	0	.	.	0	.	.	.
415	C07202	Metoprolol	0	.	.	0	.	.	.
416	C00092	Glucose 6-phosphate	0	.	.	0	.	.	.
417	C02794	3-Hydroxy-kynurenine	0	.	.	0	.	.	.
418	C00526	2'-Deoxyuridine	0	.	.	0	.	.	.
419	C00219	Arachidonic acid	0	.	.	0	.	.	.
420	C00345	6-Phosphogluconic acid	0	.	.	0	.	.	.
421	-	Juniperic acid	0	.	.	0	.	.	.
422	C06428	Eicosapentaenoic acid	0	.	.	0	.	.	.
423	C00931	Porphobilinogen	0	.	.	0	.	.	.
424	C01177	Inositol phosphate	0	.	.	0	.	.	.
425	C00214	Thymidine	0	.	.	0	.	.	.
426	C01017	5-Hydroxy-tryptophan	0	.	.	0	.	.	.
427	C19670	Oleamide	0	.	.	0	.	.	.
428	C00299	Uridine	0	.	.	0	.	.	.
429	C05659	5-Methoxytryptamine	0	.	.	0	.	.	.
430	C00526	2'-Deoxyuridine	0	.	.	0	.	.	.
431	C00449	Saccharopine	0	.	.	0	.	.	.
432	C00299	Uridine	0	.	.	1	0.00293	.	.
433	D06890	p-Aminohippuric acid	0	.	.	0	.	.	.
434	C05659	5-Methoxytryptamine	0	.	.	0	.	.	.
435	C00214	Thymidine	0	.	.	0	.	.	.
436	C00120	Biotin	0	.	.	0	.	.	.
437	C01598	Melatonin	0	.	.	0	.	.	.
438	C05382	Sedoheptulose 7-phosphate	0	.	.	0	.	.	.
439	C00449	Saccharopine	0	.	.	0	.	.	.

440	C01817	Homocystine	0	.	.	0	.	.	.
441	C00294	Inosine	1	0.000336	.	2	0.000607	0.000343	0.6357
442	-	Fendiline	0	.	.	0	.	.	.
443	C06429	Docosahexaenoic acid	0	.	.	0	.	.	.
444	C00089	Sucrose	17	0.00673	0.00515	28	0.0175	0.0275	0.1177
445	C00270	N-Acetylneuraminic acid	4	0.0011	0.000308	6	0.000751	0.000191	0.0544
446	C16513	Docosapentaenoic acid	0	.	.	0	.	.	.
447	C00212	Adenosine	0	.	.	0	.	.	.
448	C00243	Lactose	10	0.00588	0.00605	17	0.00532	0.0044	0.7839
449	-	Fendiline	0	.	.	0	.	.	.
450	C00243	Lactose	2	0.0117	0.00399	2	0.00855	0.000789	0.3892
451	C01762	Xanthosine	0	.	.	0	.	.	.
452	C13858	Batyl alcohol	0	.	.	0	.	.	.
453	C00208	Maltose	3	0.00396	0.00149	3	0.00458	0.00162	0.6523
454	C01083	Trehalose	18	0.002	0.000697	28	0.00191	0.000504	0.6352
455	C00475	Cytidine	0	.	.	0	.	.	.
456	D08266	Lactitol	3	0.0019	0.000392	6	0.00172	0.000309	0.4615
457	C00208	Maltose	5	0.00432	0.000905	3	0.00555	0.0031	0.4165
458	C00750	Spermine	0	.	.	0	.	.	.
459	C00386	Carnosine	0	.	.	0	.	.	.
460	C00387	Guanosine	0	.	.	0	.	.	.
461	-	Monostearin	1	0.000328	.	1	0.000335	.	.
462	D04845	Maltitol	0	.	.	0	.	.	.
463	C00170	5'-Methylthioadenosine	0	.	.	0	.	.	.
464	C00170	5'-Methylthioadenosine	0	.	.	0	.	.	.
465	C00252	Isomaltose	15	0.00174	0.00059	18	0.00151	0.000214	0.1206
466	C00105	Uridine monophosphate	0	.	.	0	.	.	.
467	C00252	Isomaltose	1	0.00307	.	1	0.00283	.	.
468	C00364	Thymidine monophosphate	0	.	.	0	.	.	.
469	C00130	Inosine monophosphate	0	.	.	0	.	.	.
470	C00655	Xanthosine monophosphate	0	.	.	0	.	.	.
471	C05443	Cholecalciferol	0	.	.	0	.	.	.
472	C00020	Adenosine monophosphate	0	.	.	0	.	.	.
473	C00689	Trehalose 6-phosphate	0	.	.	0	.	.	.

474	C00968	Adenosine 3',5'-cyclic monophosphate	0	.	.	0	.	.	.
475	C00187	Cholesterol	0	.	.	0	.	.	.

Blank column was below the detection limit..

Dark coloum was stastically significant .

Supplemental Table 2. Characteristics of the postmortem brain samples from Neuropathology Consortium of the Stanley Medical Research Institute.

Characteristics	Control (n=15)	MDD (n=15)	Schizophrenia (n=15)	BD (n=15)	P value
Age at death (years)	48.1 ± 10.7 (29-68)	46.5 ± 9.3 (30-65)	44.5 ± 13.1 (25-62)	42.3 ± 11.7 (25-61)	0.540[a]
Gender (male/female)	9/6	9/6	9/6	9/6	
PMI (hrs)	23.7 ± 9.95	27.5 ± 10.7	33.7 ± 14.6	32.5 ± 16.1	0.147[a]
Brain pH	6.27 ± 0.24	6.18 ± 0.21	6.16 ± 0.26	6.18 ± 0.23	0.616[a]
Brain hemispheres (right/left)	7/8	6/9	6/9	8/7	0.864[b]
Brain weight (g)	1501.0 ± 164.1	1462.0 ± 142.1	1471.7 ± 108.2	1441.2 ± 171.5	0.740[a]
Storage days	338.2 ± 234.2	434.0 ± 290.0	621.1 ± 233.1	620.5 ± 172.3	0.003[a]
Age of onset (years)		33.9 ± 13.3	23.2 ± 8.0	21.5 ± 8.4	0.003[a]
Duration of disease (years)		12.7 ± 11.1	21.3 ± 11.4	20.1 ± 9.7	0.068[a]
History of Psychosis			15	11 with (4 without)	0.100[c]
Fluphenazine equivalent (mg)			52267 ± 62062 (1 never)	20827 ± 24016 (3 never)	0.084[d]

The data are shown the mean ± SD. MDD: major depressive disorder, BD: bipolar disorder. PMI: postmortem interval.

[a]: One-way ANOVA, [b]: x^2 test for independence, [c]: Fisher’s exact test, [d]: Student t-test.
